# Long-term oncological outcome in patients with cervical cancer after 3 trimodality treatment (radiotherapy, platinum-based chemotherapy, and robotic surgery)

**DOI:** 10.1097/MD.0000000000025271

**Published:** 2021-04-02

**Authors:** Oana Gabriela Trifanescu, Laurentia Nicoleta Gales, Georgia Luiza Serbanescu, Anca Florina Zgura, Laura Iliescu, Claudia Mehedintu, Rodica Maricela Anghel

**Affiliations:** a Carol Davila University of Medicine and Pharmacy; b Department of Medical Oncology, “Prof. Dr. Al. Trestioreanu” Institute of Oncology; c Department of Medical Oncology, Oncofort Hospital; d Department of Obstetrics and Gynecology, Malaxa Clinical Hospital, Bucharest, Romania.

**Keywords:** cervical cancer, chemotherapy, radiotherapy, robotic surgery

## Abstract

Cervical cancer represents a general health issue spread all over the globe, which prompts the surge of scientific survey toward the rise of survival and condition of life of these patients. American and European guidelines suggest the open surgery, laparoscopic, and robotic surgery are the main therapeutic approaches for radical hysterectomy for patients with cervical cancer. This is the first survey to analyze the long-term oncological outcome of an extensive series of subjects cared for with multimodality treatment, here comprising robotic surgery.

This study intents to evaluate the long-term oncological result in patients diagnosed with cervical cancer treated with radiotherapy (±chemotherapy) and robotic surgery compared with open surgery. Medical files of 56 patients diagnosed with cervical cancer who underwent a robotic hysterectomy and radiotherapy ± chemotherapy were retrospectively analyzed.

The median age at diagnosis was 50.5 (range: 23–70). Eleven patients (19.6%) presented in an early stage (IB–IIA) and 80.4% advanced stage (IIB–IVA). Overall response rate after radiotherapy and chemoradiotherapy was 96.2%. Pathologic complete response was obtained in 64% of patients. After a median follow-up of 60 months (range: 6–105 months), 8 patients (14.2%) presented local recurrence or distant metastases. Disease-free survival (DFS) was 92% at 2 years and 84% at 3 and 5 years. Overall survival (OS) rates at 2, 3, and 5 years for patients with robotic surgery were 91%, 78%, and 73%, median OS not reached. OS was lower in the arm of open surgery (2, 3, and 5 years 87%, 71%, and 61%, respectively; median OS was 72 months *P* = .054). The multivariate analysis regarding the outcome of patients revealed an advantage for complete versus partial response (*P* < .002), for early versus advanced stages (*P* = .014) and a 10% gained in DFS at 3 years for patients in whom chemoradiotherapy was administered (DFS at 3 years 75% vs 85%) in patients with advanced stages.

Robotic surgery has a favorable oncological outcome when associated with multimodal therapy.

## 1 Introduction

Cervical cancer represents a general health issue spread all over the globe, which prompts the surge of scientific survey toward the rise of survival and condition of life of these patients. In most industrialized countries, the incidence of invasive cervical cancer has been decreasing in the last 30 years. It determined 2.2% of annual deaths in the USA in 2018, 3.8% of cancer deaths in women in Europe for an identical rate of 2% of deaths annually.^[1,2]^ The peak incidence of cervical cancer is in the fifth decade of life (49 years).^[3]^ As a consequence, it is a significant cause of early death because relatively young women are affected.

In modern oncology, it becomes more and more apparent that the outcome of patients with cancer is improved when using the multimodality approach. Using the most effective method in the right moment without influencing the quality of life of the patients became our goal.

Concurrent chemotherapy with radiotherapy is considered to be the standard therapeutic option for locally advanced and high-risk early-stage cervical cancers.^[4]^ Adding cisplatin to radiation therapy improves the rates of overall survival (OS) and progression-free survival among these patients.^[5,6]^ Since the early 2000s, the application of robotics to surgery has been a major technological breakthrough. It allows different types of surgery to be performed by replicating the steps of a conventional procedure but in a less invasive and therefore less traumatic manner for the patient.^[7]^ Nowadays, the development of robotic hysterectomy has simplified the use of the technics of minimally invasive techniques for the treatment of women with early or advanced and recurrent uterine cancer.^[8,9]^ The system gives the surgeon the feeling that his hands are immersed in the patient's body, even as the surgeon operates from his remote console. Such a system ensures a perfect view of the operating field through the use of 2 cameras, providing a more precise, sharper 3D vision and extreme stability. The surgeon can also, at any time, easily zoom in and move around the operating site. For patients, compared to open radical hysterectomy, robotic surgery is characterized by reduced pain, blood loss, infection, adhesions, and length of hospitalization, and a quicker return to work.^[10,11]^ There are still controversial discussions surrounding this type of surgery: cost-effectiveness research and long-term efficacy.^[12]^

The goal of the research is to appraise the long-term oncologic result in patients with cervical cancer. All patients were evaluated after multimodality treatment (robotic surgery, radiotherapy, chemotherapy), asserted as disease-free survival (DFS) and OS. Acute and late toxicity and tolerability for each treatment modality were also reported.

## 2 Materials and method

Data from medical files were retrospectively analyzed for patients with cervical cancer treated in the Oncofort Hospital between 2008 and 2014. A total of 108 patients with cervical cancer were admitted for oncological treatment after surgery. Fifty-six patients underwent a robotic hysterectomy, and 52 patients had open surgery (control arm).

### 2.1 Patient selection criteria

Eligibility criteria for patients included were consecutive patients admitted in our institute for treatment (radiotherapy or chemotherapy) with a histopathological report of the cervix malignancy.

At the beginning of the treatment, patients had an ECOG performance status 0–2, without significant comorbidities. All subjects had reasonable bone marrow reserve showed by absolute neutrophils count, normal hepatic and renal function, and standard images on chest computed tomography (CT). All the included patients received a gynecological examination. For tumor assessment, CT with contrast medium of abdomen and pelvis was used.

### 2.2 Treatment

All patients included in our study had cervical cancer confirmed by biopsy. After confirmation of the malignancy, patients underwent surgery as the first step—3 of 56 (5.4%) or were managed with neoadjuvant therapies comprising radiotherapy and chemotherapy—53/56 (94.6%).

Radiotherapy was performed according to the treatment plan on the tumor bed or over nodes for operated patients and for those who underwent only the biopsy, 5 days a week. The primary cervical tumor was then boosted, using high-dose brachytherapy, with an additional 15 Gy.

In patients with a tumor larger than 4 cm in diameter or with parametrial infiltration, concomitant chemotherapy with cisplatin (40 mg/m^2^ weekly) was added. We faced patient refusal or contraindication to cisplatin chemotherapy in 37.5% of cases. Postoperative chemotherapy was administered in patients with residual disease after neoadjuvant treatment (radiotherapy ± chemotherapy). All chemotherapy regimens administered were platinum based using the following combinations: 5-fluorouracil with cisplatin every 21 days, paclitaxel with cisplatin or carboplatin every 21 days for patients with impaired renal function, or topotecan with cisplatin every 21 days.

Follow-up visits were planned every 3 months after surgery with clinical and laboratory assessment, cervical/vaginal cytology annually, imaging (chest radiography, CT, magnetic resonance imaging) as indicated based on symptoms or examination findings suspicious for recurrence.

The surgical technique included minimal invasive methods. Robotic bilateral periaortic lymphadenectomy from the common iliac artery to the inferior boundary of the circumflex iliac vein, hysterectomy ± bilateral salpingo-oophorectomy, and peritoneal lavage was performed in 56 cases. The DaVinci S robotic system was used in a single experienced surgical center. The controlled arm underwent the same procedure but with open surgery.

### 2.3 Statistical analysis

The statistical analysis was accomplished using SPSS. The following points were followed: DFS and OS. DFS and OS data were calculated using the Kaplan-Meyer procedure to study survival or risk. Pearson test was employed to analyze the rapport between categorical variables. The significance point was established at *P* < .05 for all statistical analyses.

## 3 Results

Between 2008 and 2014, 108 patients diagnosed with cervical cancer were admitted for treatment. In 56 patients, robotic hysterectomy was performed, and 52 were operated in a classical manner (control group). The median age at diagnosis was 50.5 years (range: 23–70 years). The vast majority of patients came from urban areas. Vaginal bleeding was the presenting symptom in almost 90% of cases.

The distribution of disease's stage at presentation was as follows: 9 patients (16.1%) stage IB, 2 patients (3.6%) stage II A, 23 patients (41.1%) stage IIB, 1 patient (1.8%) stage IIIA, 14 patients (25%) with stage IIIB, and 5 (8.9%) patients with stage IVA and 2 patients (3.6%) stage IVB.

**Figure 1:**
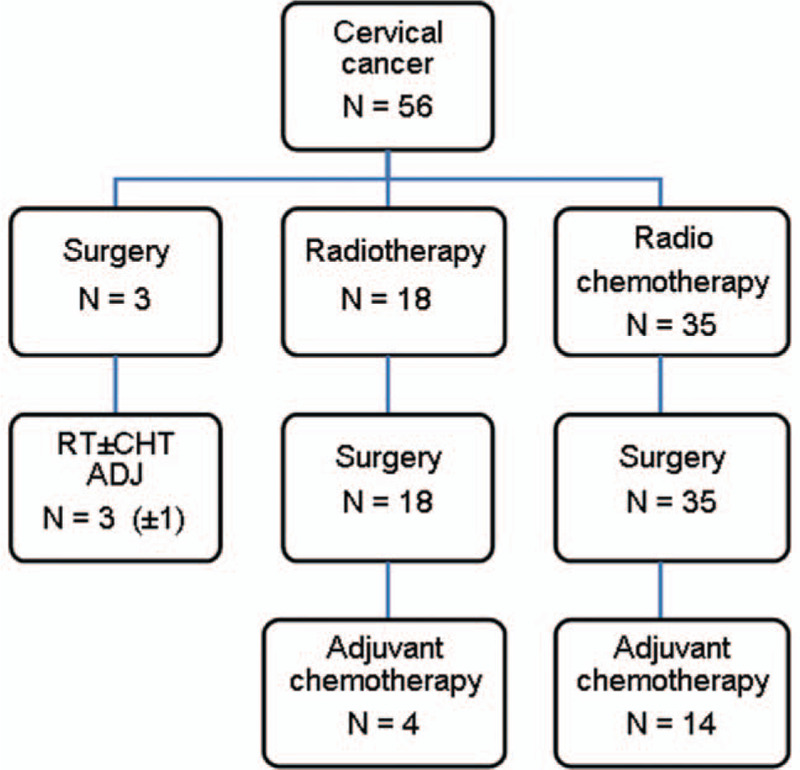
Multimodality treatment sequence in studied patients.

The 56 patients were managed as follows: 3 were operated as the first step, 35 underwent radiochemotherapy, and in 18 patients, radiotherapy was the only treatment before surgery (Fig. 1).

In patients who underwent neoadjuvant treatment (radiotherapy ± chemotherapy), 35 of 53 (66%) had a complete pathological response and 18 of 53 (34%) had residual disease present at the time of surgery. Patients with the residual disease were treated with adjuvant chemotherapy.

In our group of patients, the mean dose of radiotherapy administered was 59.06Gy (range 39.8–89.4 Gy), and a median dose of 60 Gy, was applied to the pelvis. The average extent of radiotherapy was 44.8 days (without brachytherapy). There is a high statistical significance regarding the rate of complete pathological response and the duration of radiotherapy. The meantime of radiotherapy was 43 days in patients with complete pathological response versus 55 days in patients without complete response (*P* = .02). The result emphasizes the importance of radiotherapy intensity dose and duration and underlines the need for another local treatment. Thirty-five patients received at least 4 administrations of concurrent chemoradiotherapy (CCRT) with cisplatin. Eighteen patients received adjuvant chemotherapy for residual disease. The most frequent adjuvant regimen was 5-fluorouracil and a platinum salt (8/18) and paclitaxel + carboplatin (8/18). The mean number of cycles administrated was 4 (limits 1–6). The overall response rate to radiotherapy and chemoradiotherapy was 94.6%.

The main symptoms acknowledged by the patients were cystitis (2/56) and rectitis (3/56), easily managed with symptomatic treatment. Grade 3 or higher for hematologic toxicity was represented by leucopenia in 4 patients (1 death caused by neutropenia postadjuvant chemotherapy), thrombocytopenia 2 of 56, and anemia 11 of 56.

**Figure 2:**
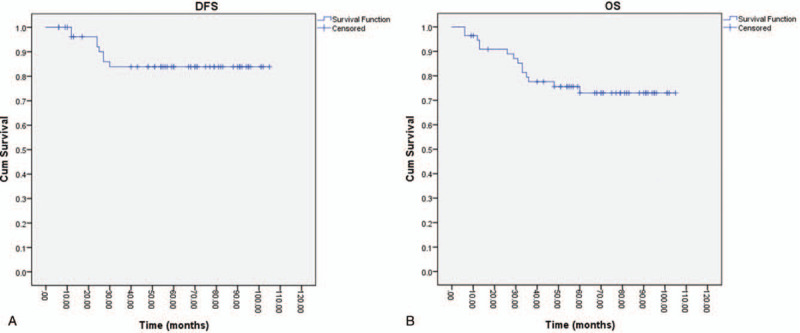
Disease-free survival (A) and overall survival (B) analysis in studied patients.

After a median follow-up of 60 months (range 6–106 months), 8 patients (14.3%) presented local recurrence in the pelvis or distant metastases, 5 patients presented with local recurrence, 3 cases with distant metastases (2 osseous metastases and 3 lymphatic and peritoneal metastases). DFS was 92% at 2 years and 83% at 3, and 5 years. Concerning the OS rates, at 2, 3, and 5 years, 91%, 78%, and 73%, respectively were alive (Fig. 2).

**Figure 3:**
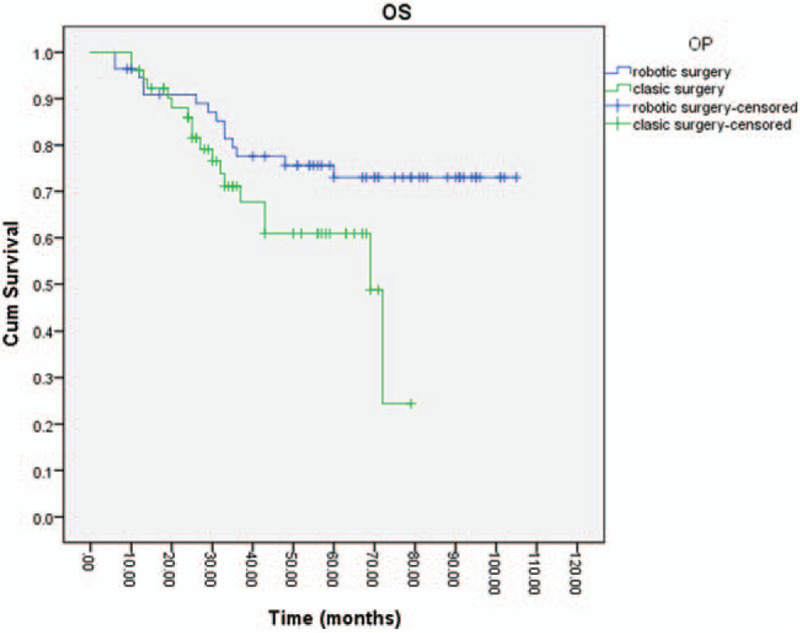
Overall survival of the open surgery group compared with the robotic surgery group.

Fourteen deaths (from the total of 108 patients) were recorded in our study population. Still, 4 patients had a no-cancer-related end, 1 patient died of an adverse event during chemotherapy, and 1 patient died because of another neoplasia. OS was lower in the group of open surgery with OS at 2, 3, and 5 years of 87%, 71%, and 61%, respectively (median OS was 72 months; *P* = .054) compared to robotic surgery (Fig. 3).

Comparing the early-stage patients (IB–IIA) (19.6%) with advanced stage (IIB–IVA) (81.4%) there was a statistical difference regarding the outcomes: in the early stage there was no recurrence (DFS at 36 months 100%), but in the advanced stage DFS at 36 months was significantly lower (DFS = 80%), *P* = .014.

**Figure 4:**
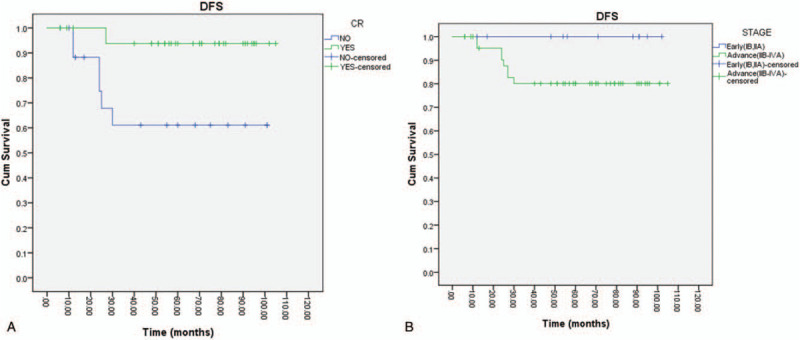
Prevalence for complete response versus partial response. A, Better results in patients with complete response versus residual disease after neoadjuvant treatment. B, Better results for early-stage (IB-IIA) versus advanced stage (IIB-IVA).

The multivariate analysis regarding the patients’ outcomes revealed a higher prevalence for complete response versus partial response (hazard ratio [HR] = 0.11; *P* < .0001; 95% confidence interval [CI] 0.001–0.357) (Fig. 4). Consistently, data showed a 10% gain in DFS at 3 years for patients in which chemoradiotherapy was administered (DFS at 3 years 75% vs 85%) in patients with advanced stages. Tumor grading (G1 vs G2 and G3) was a prognostic marker (*P* = .011). No statistical difference was noted between the outcomes of postoperative treatments (adjuvant chemotherapy) underlining once more the importance of the complete response after chemoradiotherapy in these patients.

## 4 Discussions

To our knowledge, this is the first survey to analyze the long-term oncological outcome of an extensive series of subjects cared for with multimodality treatment, here comprising robotic surgery. Continuing oncological effects of robotic surgery in uterine malignancy are not well settled. Feasibility and safety of robotic surgery for gynecological oncology are demonstrated only by controlled clinical trials, and randomized controlled trials are scarce.^[13]^

Historically, local control rate was ranging between 88% and 95% for stage IB, 70% to 80% for stage IIB, and 30% to 40% for stage III and 5 years survival was more than 80% for stage IB, 65% for stage IIB, and 40% for stage III.^[14,15]^ The present study demonstrates that at 3 years, there was no recidivate in patients with stage IB and IIA, DFS for stage IIB was 92%, for stage III, 43%, and for stage IV 52%. The overall DFS was 90% at 2 years and 84% at 3 years. Moreover, the study population comprised mostly of cases with advanced disease (72.7%)—subjects with stage IIB to IVA. These results were obtained without a significant increase in toxicity rates using multimodality treatment (radiochemotherapy and robotic surgery).

In terms of short-term surgical results, the safeness and feasibility of robotic surgery in uterine malignancy were well confirmed, and many studies indicated a diminishing of blood loss, briefer hospital stay, and promptly restoration.^[16–18]^ Surgery length, blood perdition, hospitalization, and aggravations were reported to be comparable in women with locally advanced cervical malignancy and those with early-stage disease. All subjects experienced radical robotic hysterectomy with pelvic lymphadenectomy succeeding neoadjuvant chemotherapy.^[19]^ The need for intravenous opioids was significantly less in robotic surgery versus open surgery.^[20]^ Such advantages are particularly important in medically ill patients with high anesthetic risk due to old age, obesity, and severe comorbidities. In a series of 235 patients with endometrial and cervical cancer, there were no differences in terms of operative times, blood loss, intra- and postoperative complications, conversion rate, and hospitalization between patients with score ASA 1–2 (n = 169) versus those with ASA score ≥3 (n = 66). Preservation of fertility and achieving pregnancy in some young women undergoing radical robotic trachelectomy is another advantage of the method.^[17]^

Patients with locally progressive cervical cancer (large tumors, parametrial invasion, suspected lymph node involvement, previous uterine fibroma) received chemoradiotherapy at first. In these subjects who were not candidates for surgery as the first treatment, to attain the best local control and to decrease the risk of distant metastases, chemoradiotherapy was followed by robotic hysterectomy.

Since 1999, multiple studies have been demonstrated the superiority of CCRT compared with conventional radiotherapy in terms of DFS and OS, with an absolute 30% to 50% decrease in the risk of death, and 6% improvement in 5 years survival rates (HR = 0.81, *P* < .001) reported in a meta-analysis published in 2008.^[6,21–24]^ In the studied patients, adding chemotherapy to radiotherapy in cases with locally advanced cervical cancer raised by 5% the 3 years DFS rate (85% vs 80%).

The possibility of using adjuvant hysterectomy after chemoradiotherapy was considered. Early studies from MD Anderson Cancer Center have shown that local recurrence rates for patients with stage IB cancers decreased when radiotherapy was followed by adjuvant hysterectomy.^[25]^ The GOG completed a prospective randomized trial of irradiation with or without adjuvant extra fascial hysterectomy in patients with stage IB tumors, 4 cm or more in diameter. Results of this trial were reported in 2003 by Keys et al^[26]^; the study demonstrated that there is no significant improvement related to survival rate in case of patients who performed an adjuvant hysterectomy (relative risk of death, 0.89; 90% CI, 0.65, 1.21).

Recently, based on some new data, the issue is debatable. A study published in 2014 evaluated the surgical morbidity and oncological results after CCRT followed by completion surgery for advanced cervical cancer (IIB–IVA). Adding classical surgery to chemoradiotherapy resulted in a decreased rate of recurrence (16.7% vs 31.7%) and improved the 3 years survival rates (72.2% vs 45.9%).^[27]^

Another recent study showed higher OS and progression-free survival rates in patients FIGO stage IIB cervical carcinoma, treated by CCRT followed by radical surgery in comparison with radical radiotherapy associated with concurrent chemotherapy (3-year OS, 94.9% vs 84.6%, *P* = .011; 3 years progression-free survival, 91.0% vs 81.8%, *P* = .049, respectively).^[28]^ Using minimally invasive surgery should result in similar good outcomes, but with less toxicity.

Due to locally advanced disease and large tumors found in the study (80%), as well as the delayed access to radiotherapy, the median dose of radiotherapy was 60 Gy, and a new surgical procedure was available, robotic hysterectomy was planned for these patients. The response rate was extremely high (94.6%), so all 53 patients were candidates for robotic hysterectomy after initial treatment. Another problem was that the patients presented with large tumors, and despite an adequate response rate, there were 19 patients with residual disease. Which is the best option for these patients, and the follow-up algorithm in the absence of surgery, is still a problem of debate, so surgery seems a realistic solution.

Ensuing the lesson learned from breast cancer, complete pathological response (64.3%) appears to be in our series, the most significant prognostic factor in patients with cervical cancer who went through radiochemotherapy. The results after surgery showed that 35.7% had residual disease after initial treatment. These patients were candidates for adjuvant chemotherapy. Unfortunately, adjuvant chemotherapy was not sufficient to improve the prognostic. In conclusion, more aggressive therapy before surgery provides a much better outcome for the patients. An essential point of view is the fact that after radiotherapy or chemoradiotherapy, the postoperative complications are more frequent, recovery is slower, and generally, the patients show hematological toxicity. Thus, it is vital for this group of patients, to waste a small amount of blood, and to recover as quickly as possible. Robotic surgery provides that outcome. In the studied patients, there was no death related to the operation, and no significant morbidity reported.

Lymph node status and the number of lymph nodes involved are some of the most significant prognostic factors. In 1 study, the 5-year survival rates of patients with stage IB and IIA without and with lymph node metastasis were 88% to 95% and 51% to 78%, respectively.^[29]^ In another study, 42 patients with early-stage cervical carcinoma underwent robotic-assisted radical hysterectomy; the median lymph node count was 25, and positive lymph nodes were identified in 12% of patients.^[18]^

In this set of patients, the mean number of lymph nodes removed was 22, and thanks to the aggressive perioperative treatment, only in 6 of 56 (10.7%) patients, lymph nodes were positive. We could not reach a definite conclusion upon the results of patients with lymph node invasion, as these cases were only a few.

In a meta-analysis issued in 2008, relapse and OS rates in patients with early-stage cervical cancer were comparable in patients subjected to radical laparoscopic hysterectomy and those who went through open laparotomy. Still, there was no information for subjects operated by robotic surgery.^[30]^

In a series of 35 patients (cervical cancer n = 19, endometrial cancer n = 16), followed-up for 20 to 22 months, the recurrence rate was 1 of 19 for cervical cancer and 2 of 16 for endometrial cancer.^[31]^

In another small group of 11 patients with occult invasive cervical cancer or local recurrence of endometrial cancer, the relapse quota after radical robotic parametrectomy and pelvic lymphadenectomy was 1 of 11 (19 months median follow-up).^[32,33]^ These data are compatible with the results that emerged from our group of patients and former experience.^[34]^

Two prospective studies were published recently. First enrolled 2461 cervical cancer patients with stage IA2–IB1, half of the patients performed minimally invasive surgery and the other half open surgery. Median follow-up was 45 months, and the 4-year mortality was 9.1% among women who performed minimally invasive surgery and 5.3% among those who performed open surgery (HR = 1.65; 95% CI, 1.22–2.22; *P* = .002 by the log-rank test).^[35]^ Another survey comprised 319 women with stage IA1–IB1 disease to minimally invasive surgery and 312 patients for open surgery. It stated that minimally invasive surgery was correlated with a lower rate of DFS than open surgery (3-year rate, 91.2% vs 97.1%; HR for disease relapse or demise from cervical cancer, 3.74; 95% CI, 1.63–8.58).^[36]^

Because of the rapid recovery after robotic surgery and the use of tree modality treatment, anxiety of patients related to disease can be diminished.^[37]^

The limitation of the study was the small number the patients treated with robotic surgery and longer follow-up needed.

## 5 Conclusions

The 3-modality treatment (radiotherapy, chemotherapy, and robotic surgery) provides excellent local control in patients with cervical cancer, especially in the early stages, without a significant increase in toxicity rates. Complete pathologic response after chemoradiotherapy offers the best chance of survival for these patients. Robotic surgery has a favorable oncological outcome when associated with multimodal therapy (radiotherapy ± chemotherapy).

Due to the advanced local stage at presentation, we intend to emphasize the importance of surgery in general and robotic surgery in particularly after chemoradiotherapy, because, in daily practice, we encounter an increasing number of recurrence after initial treatment, even in patients who achieved complete response.

Further multicenter randomized studies are needed to prove either equivalence or superiority of robotic surgery comparative with traditional open surgery's results.

## Author contributions

**Conceptualization:** Oana Gabriela Trifanescu, Laurentia Nicoleta Gales, Rodica Maricela Anghel.

**Data curation:** Oana Gabriela Trifanescu, Laurentia Nicoleta Gales, Georgia Luiza Serbanescu.

**Formal analysis:** Georgia Luiza Serbanescu.

**Investigation:** Oana Gabriela Trifanescu.

**Methodology:** Laurentia Nicoleta Gales.

**Resources:** Claudia Mehedintu.

**Supervision:** Zgura Anca, Rodica Maricela Anghel.

**Writing – review & editing:** Zgura Anca, Laura Iliescu, Claudia Mehedintu.

## References

[1] Arbyn M Castellsagué X De Sanjosé S. Worldwide burden of cervical cancer in 2008. Ann Oncol 2011;22:2675–86. 21471563 10.1093/annonc/mdr015

[2] Yavari K. Anti-angiogenesis therapy of cancer cells using 153Sm-Bevasesomab. Emerg Sci J 2018;2:130–9.

[3] Available at: https://seer.cancer.gov [accessed May 18, 2019].

[4] Petignat P. Diagnosis and management of cervical cancer. BMJ 2007;335:765–8. 17932207 10.1136/bmj.39337.615197.80 PMC2018789

[5] Green JA Kirwan JM Tierney JF. Survival and recurrence after concomitant chemotherapy and radiotherapy for cancer of the uterine cervix: a systematic review and meta-analysis. Lancet 2001;358:781–6. 11564482 10.1016/S0140-6736(01)05965-7

[6] Peters WA 3rd Liu PY Barrett RJ 2nd. Concurrent chemotherapy and pelvic radiation therapy compared with pelvic radiation therapy alone as adjuvant therapy after radical surgery in high-risk early-stage cancer of the cervix. J Clin Oncol 2000;18:1606–13. 10764420 10.1200/JCO.2000.18.8.1606

[7] Wright JD Burke WM Wilde ET. Comparative effectiveness of robotic versus laparoscopic hysterectomy for endometrial cancer. J Clin Oncol 2012;30:783–91. 22291073 10.1200/JCO.2011.36.7508 PMC3295567

[8] Lau S Vaknin Z Ramana-Kumar AV. Outcomes and cost comparisons after introducing a robotics program for endometrial cancer surgery. Obstet Gynecol 2012;119:717–24. 22433334 10.1097/AOG.0b013e31824c0956

[9] Smith AL Pareja R Ramirez PT. Robotic radical hysterectomy. A literature review. Minerva Ginecol 2009;61:339–46. 19745798

[10] Fanning J Hojat R Johnson J. Robotic radical hysterectomy. Minerva Ginecol 2009;61:53–5. 19204661

[11] Farache C Alonso S Ferrer Marsollier C. Robotic surgery in gynecologic oncology: retrospective and comparative study with laparotomy and laparoscopy. J Gynecol Obstet Biol Reprod (Paris) 2012;41:353–62. 22542372 10.1016/j.jgyn.2012.03.004

[12] Krill LS Bristow RE. Robotic surgery: gynecologic oncology. Cancer J 2013;19:167–76. 23528726 10.1097/PPO.0b013e31828a3293

[13] Liu H Lawrie TA Lu D. Robot-assisted surgery in gynaecology. Cochrane Database Syst Rev 2014;12:CD011422. 10.1002/14651858.CD011422 PMC6457792 25493418

[14] Barillot I Horiot JC Pigneux J. Carcinoma of the intact uterine cervix treated with radiotherapy alone: a French cooperative study: update and multivariate analysis of prognostics factors. Int J Radiat Oncol Biol Phys 1997;38:969–78. 9276361 10.1016/s0360-3016(97)00145-4

[15] Perez CA Grigsby PW Chao KS. Tumor size, irradiation dose, and long-term outcome of carcinoma of uterine cervix. Int J Radiat Oncol Biol Phys 1998;41:307–17. 9607346 10.1016/s0360-3016(98)00067-4

[16] Lowe MP Chamberlain DH Kamelle SA. A multi-institutional experience with robotic-assisted radical hysterectomy for early stage cervical cancer. Gynecol Oncol 2009;113:191–4. 19249082 10.1016/j.ygyno.2009.01.018

[17] Nick AM Frumovitz MM Soliman PT. Fertility sparing surgery for treatment of early-stage cervical cancer: open vs. robotic radical trachelectomy. Gynecol Oncol 2012;124:276–80. 22035808 10.1016/j.ygyno.2011.09.035 PMC4286385

[18] Yim GW Kim YT. Robotic surgery in gynecologic cancer. Curr Opin Obstet Gynecol 2012;24:14–23. 22123220 10.1097/GCO.0b013e32834daebc

[19] Vitobello D Siesto G Pirovano C. Surgical outcomes of robotic radical hysterectomy after neoadjuvant chemotherapy for locally advanced cervical cancer: comparison with early stage disease. Eur J Surg Oncol 2013;39:87–93. 23122543 10.1016/j.ejso.2012.10.001

[20] Soliman PT Langley G Munsell MF. Analgesic and antiemetic requirements after minimally invasive surgery for early cervical cancer: a comparison between laparoscopy and robotic surgery. Ann Surg Oncol 2013;20:1355–9. 23054117 10.1245/s10434-012-2681-z PMC4264594

[21] Keys HM Bundy BN Stehman FB. Cisplatin, radiation, and adjuvant hysterectomy compared with radiation and adjuvant hysterectomy for bulky stage IB cervical carcinoma. N Engl J Med 1999;340:1154–61. 10202166 10.1056/NEJM199904153401503

[22] Morris M Eifel PJ Lu J. Pelvic radiation with concurrent chemotherapy compared with pelvic and para-aortic radiation for high-risk cervical cancer. N Engl J Med 1999;340:1137–43. 10202164 10.1056/NEJM199904153401501

[23] Rose PG Bundy BN Watkins EB. Concurrent cisplatin-based radiotherapy and chemotherapy for locally advanced cervical cancer. N Engl J Med 1999;340:1144–53. 10202165 10.1056/NEJM199904153401502

[24] Whitney CW Sause W Bundy BN. Randomized comparison of fluorouracil plus cisplatin versus hydroxyurea as an adjunct to radiation therapy in stage IIB-IVA carcinoma of the cervix with negative para-aortic lymph nodes: a Gynecologic Oncology Group and Southwest Oncology Group study. J Clin Oncol 1999;17:1339–48. 10334517 10.1200/JCO.1999.17.5.1339

[25] Durrance FY Fletcher GH Rutledge FN. Analysis of central recurrent disease in stages I and II squamous cell carcinomas of the cervix on intact uterus. Am J Roentgenol Radium Ther Nucl Med 1969;106:831–8. 10.2214/ajr.106.4.831 5806012

[26] Keys HM Bundy BN Stehman FB. Gynecologic Oncology Group. Radiation therapy with and without extrafascial hysterectomy for bulky stage IB cervical carcinoma: a randomized trial of the Gynecologic Oncology Group. Gynecol Oncol 2003;89:343–53. 12798694 10.1016/s0090-8258(03)00173-2

[27] Sun L Sheng X Jiang J. Surgical morbidity and oncologic results after concurrent chemoradiation therapy for advanced cervical cancer. Int J Gynaecol Obstet 2014;125:111–5. 24548892 10.1016/j.ijgo.2013.07.041

[28] Wang N Li WW Li JP. Comparison of concurrent chemoradiotherapy followed by radical surgery and high-dose-rate intracavitary brachytherapy: a retrospective study of 240 patients with FIGO stage IIB cervical carcinoma. Onco Targets Ther 2014;7:91–100. 24421644 10.2147/OTT.S52710 PMC3888351

[29] Landoni F Maneo A Colombo A. Randomised study of radical surgery versus radiotherapy for stage Ib-IIa cervical cancer. Lancet 1997;350:535–40. 9284774 10.1016/S0140-6736(97)02250-2

[30] Ramirez PT Schmeler KM Soliman PT. Fertility preservation in patients with early cervical cancer: radical trachelectomy. Gynecol Oncol 2008;110:S25–8. 18501409 10.1016/j.ygyno.2008.03.025

[31] Kimmig R Aktas B Buderath P. Definition of compartment-based radical surgery in uterine cancer: modified radical hysterectomy in intermediate/high-risk endometrial cancer using peritoneal mesometrial resection (PMMR) by M Höckel translated to robotic surgery. World J Surg Oncol 2013;11:198. 23947937 10.1186/1477-7819-11-198 PMC3751733

[32] Rodica Maricela A Minea LN Bacinschi X. Concomitant radical non-platinum radiochemotherapy for elderly patients with invasive bladder cancer. J Clin Oncol 2009;15 suppl:e16135.

[33] Vitobello D Siesto G Bulletti C. Robotic radical parametrectomy with pelvic lymphadenectomy: our experience and review of the literature. Eur J Surg Oncol 2012;38:548–54. 22425283 10.1016/j.ejso.2012.02.188

[34] Anghel R Minea L Bacinschi X. Cisplatin-based radiochemotherapy in elderly patients with cervical cancer. J Clin Oncol 2010;28: 15 suppl: e15550.

[35] Melamed A Margul DJ Chen L. Survival after minimally invasive radical hysterectomy for early-stage cervical cancer. N Engl J Med 2018;379:1905–14. 30379613 10.1056/NEJMoa1804923 PMC6464372

[36] Ramirez PT Frumovitz M Pareja R. Minimally invasive versus abdominal radical hysterectomy for cervical cancer. N Engl J Med 2018;379:1895–904. 30380365 10.1056/NEJMoa1806395

[37] Popa-Velea O Bacinschi X. Dynamics of anxiety in cervical cancer patients undergoing radiotherapy: preliminary results. J Psychosom Res 2019;121:135.

